# Multiple Sclerosis: Shall We Target CD33?

**DOI:** 10.3390/genes11111334

**Published:** 2020-11-12

**Authors:** Vasileios Siokas, Zisis Tsouris, Athina-Maria Aloizou, Christos Bakirtzis, Ioannis Liampas, Georgios Koutsis, Maria Anagnostouli, Dimitrios P. Bogdanos, Nikolaos Grigoriadis, Georgios M. Hadjigeorgiou, Efthimios Dardiotis

**Affiliations:** 1 Laboratory of Neurogenetics, Department of Neurology, University Hospital of Larissa, Faculty of Medicine, School of Health Sciences, University of Thessaly, 41110 Larissa, Greece; vsiokas@med.uth.gr (V.S.); zitsouri@med.uth.gr (Ζ.Τ.); aaloizou@med.uth.gr (A.-M.A.); liampasioannes@gmail.com (I.L.); hadjigeorgiou.georgios@ucy.ac.cy (G.M.H.); 2 Multiple Sclerosis Center, B’ Department of Neurology, AHEPA University Hospital, Aristotle University of Thessaloniki, GR54636 Thessaloniki, Greece; bakirtzischristos@yahoo.gr (C.B.); grigoria@med.auth.gr (N.G.); 3 Neurogenetics Unit, 1st Department of Neurology, Eginition Hospital, School of Medicine, National and Kapodistrian University of Athens, Vassilissis Sofias 72-74 Ave, 11528 Athens, Greece; gkoutsis@med.uoa.gr; 4 Multiple Sclerosis and Demyelinating Diseases Unit and Immunogenetics Laboratory, 1st Department of Neurology, Eginition Hospital, School of Medicine, National and Kapodistrian University of Athens, 115 28 Athens, Greece; managnost@med.uoa.gr; 5 Department of Rheumatology and Clinical Immunology, Faculty of Medicine, School of Health Sciences, University of Thessaly, 41110 Larissa, Greece; bogdanos@uth.gr; 6 Department of Neurology, Medical School, University of Cyprus, 1678 Nicosia, Cyprus

**Keywords:** multiple sclerosis, CD33, single nucleotide polymorphism, genetics, rs3865444

## Abstract

Background: Multiple sclerosis (MS) is a chronic disease of the central nervous system (CNS). Myeloid lineage cells (microglia and macrophages) may participate in the pathogenic mechanisms leading to MS. CD33 is a transmembrane receptor, mainly expressed by myeloid lineage cells. CD33 rs3865444 is a promoter variant previously associated with Alzheimer’s disease, whose role in MS remains obscure. Objective: To assess the role of CD33 rs3865444 in MS risk. Methods: We genotyped 1396 patients with MS and 400 healthy controls for the presence of the CD33 rs3865444 variant. Odds ratios (ORs) with the respective 95% confidence intervals (CIs), were calculated with the SNPStats software, assuming five genetic models (co-dominant, dominant, recessive, over-dominant, and log-additive), with the G allele as the reference allele. The value of 0.05 was set as the threshold for statistical significance. Results: CD33 rs3865444 was associated with MS risk in the dominant (GG vs. GT + TT; OR (95% C.I.) = 0.79 (0.63–0.99), *p* = 0.041) and the over-dominant (GG + TT vs. GT; OR (95% C.I.) = 0.77 (0.61–0.97), *p* = 0.03) modes of inheritance. Given that the GG genotype was more frequent and the GT genotype was less frequent in MS patients compared to controls—while the observed frequency of the TT genotype did not differ between the two groups—the observed difference in MS risk may be stemming from either the GG (as a risk factor) or the GT (as a protective factor) genotype of CD33 rs3865444. Conclusions: Our preliminary results suggest a possible contribution of CD33 rs3865444 to MS. Therefore, larger multiethnic studies should be conducted, investigating the role of CD33 rs3865444 in MS.

## 1. Introduction

Multiple sclerosis (MS) is a chronic disease of the central nervous system (CNS). Epidemiological data suggest that MS is commonest among women, peaks, incidence-wise, during the third decade of life, and is the main cause of neurological disability during adulthood [1]. Regarding MS frequency, more than two million individuals suffer from MS throughout the world [2].

The genetic component of MS is indisputable [2]. The evidence for a genetic background of MS emerges from quite a few scientific sources. As a matter of fact, siblings of affected individuals with MS appear to have a higher risk of developing MS (up to 20-fold), compared to the general population. Additionally, this risk is further increased (up to 150-fold) in monozygotic twins [3]. Secondly, accumulating epidemiological data reveal higher MS rates in distinct ancestral groups (e.g., individuals in north Europe and North America present an elevated MS incidence in comparison with other populations) [1]. On the other hand, differences in MS incidence between specific geographical regions have been attributed to distinctive exposure to various environmental factors [4]. The latter, in combination with the fact that even in identical twins the relative MS risk does not reach 100%, denotes the contribution of additional risk factors in MS [5]. 

Overall, the genetic architecture of MS follows as complex model, which can be described as polygenic, following a non-Mendelian pattern [6]. According to this model, a “common variant–common disease” paradigm of genetic influences and inheritance is present, and common risk alleles determine (individually) a moderate portion of MS risk [6]. However, this model can only explain 20% of MS risk heritability, with nearly 5% of MS heritability being interpreted by coding low-frequency variants, suggesting an even more complex pattern of MS genetics [7,8].

Research on the pathophysiology of MS continues to be challenging. Inflammation, neuro-axonal degeneration, glutamate excitotoxicity, ionic imbalance, increased sodium levels energy failure, and astrocyte and microglia activation are among the pathogenic mechanisms leading to MS [9,10,11,12]. CD33, also known as Siglec-3 (sialic acid-binding Ig-like lectin 3) is a transmembrane receptor and a member of the Siglec family of immunomodulatory receptors, mainly expressed on myeloid lineage cells, such as microglia and macrophages [13,14]. Microglia constitute almost 40% of early MS lesion’s mass and are the inflammation primers during all phases of MS lesion formation [15]. Macrophages dominate CNS infiltration and are crucial for inflammation in the acute MS phase [16]. As such, both macrophages and microglia are of great importance in MS, mainly regarding inflammation, while they also remain in a chronic state of activation during MS course [17]. CD33 can also be found in some lymphoid lineage cells [13,14]. Therefore, alterations in CD33 may lead to defective functions of all types of immune system cells [18] and to loss of immune tolerance, defective remyelination, and faulty control of pro-inflammatory cytokines production, thus predisposing to MS.

A frameshift variant in the CD33 gene has emerged as a potential risk locus for neuromyelitis optica spectrum disorder (NMOSD) [18], a disease that shares some similarities with MS [19]. Moreover, from a genetic point of view, CD33 rs3865444 is a common promoter variant, which appears to modify CD33 function and has also been previously associated with the commonest neurodegenerative disease, i.e., Alzheimer’s disease (AD) [20,21]. 

Taking into account the former considerations, namely, a) that genetics indisputably influences MS risk in a complicated manner, b) that CD33 may be implicated in some cardinal pathways of microglia and macrophage functions (mainly) and thus possibly in MS, c) that a CD33 variant has been associated with NMOSD, and d) that the CD33 rs3865444 is a promoter variant that appears to modify CD33 function and has been associated with AD, we aimed to examine the possible crude association of rs3865444 with MS by performing a case-control study.

## 2. Materials and Methods 

### 2.1. Participants

One thousand three hundred ninety-six (1396) MS cases and 400 healthy controls were recruited from three MS centers in Greece: (a) the University Hospital of Larissa (University of Thessaly) in Central Greece, (b) the Eginition University Hospital of the National and Kapodistrian University of Athens Medical School, and (c) the AHEPA University Hospital of the Aristotle University in Thessaloniki in North Greece. The diagnosis of MS was performed by a senior neurologist following the 2017 McDonald criteria [22]. All participants provided written informed consent. The study protocol received the approval of the local ethics committee (University Hospital of Larissa: 57236/15-12-2014). The main features of MS patients and healthy controls have been previously described [23].

### 2.2. Molecular Genetics

With the method of salting out, we isolated genomic DNA from peripheral blood leucocytes [24,25]. Genotyping of rs3865444 was performed using the following pair of flanking primers and probes: allele-flanking primers: F: 5′-GAGCTTAAAGACTGGCTTTCTGA-3′, R:5′-TCCAAGTCGCTTCCATTTTC-3′, allele-specific primers: F: 5′-GATAAGAATGAAGAAAAAAGAATAAAAAAG-3′, R1: 5′-TCTCCAGAGGTTTTGTTTGCTC-3′, R2: 5′-TCTCCAGAGGTTTTGTTTGCTT. The following conditions were used for amplification: 50 °C for 2 min, 95 °C for 10 min, followed by 40 cycles at 95 °C for 15 s and 60 °C for 1 min. All patients with MS and the healthy controls were genotyped for CD33 rs3865444 by applying TaqMan allele-specific discrimination assays using an ABI PRISM 7900 Sequence Detection System. The results were analyzed with the SDS software (Applied Biosystems, Foster City, CA, USA). The genotype call rate counted equal to 97.33%.

### 2.3. Statistical Analysis

Using the CaTS Power Calculator (http://csg.sph.umich.edu/abecasis/cats/), we calculated the power of our analysis [26]. The power of our sample, with a minor allele frequency of 24% in the MS cohort, was 80.2%, with a genetic risk equal to 1.26. The Hardy–Weinberg equilibrium (HWE) in healthy controls and MS cases and the test for association (odds ratios (ORs) with the respective 95% confidence intervals (CIs)) were calculated with the SNPStats software (https://www.snpstats.net/) [27], assuming five genetic models (co-dominant, dominant, recessive, over-dominant, and log-additive), with the G allele as the reference allele. The value of 0.05 was set as the threshold for statistical significance.

## 3. Results

The clinical characteristics of MS and healthy control groups are presented in Table 1.

Healthy controls were in HWE (*p* > 0.05), while MS cases were not (*p* < 0.05). The frequencies of genotypes and alleles of the CD33 rs3865444 variant in the entire sample, in MS cases and in healthy controls, are shown in Table 2. Analysis of the genotypic frequencies of CD33 rs3865444 showed that the GG, GT, and TT genotypes were found in 54%, 39%, and 7% of the healthy controls, respectively, while the frequencies of GG, GT, and TT for the individuals with MS were 60%, 33%, and 7%, respectively.

Single-locus analysis revealed a statistically significant association between risk of MS and the presence of CD33 rs3865444 in the dominant (GG vs. GT + TT; OR (95% C.I.) = 0.79 (0.63–0.99), *p* = 0.041) and the over-dominant (GG + TT vs. GT; OR (95% C.I.) = 0.77 (0.61–0.97), *p* = 0.03) modes of inheritance. The ORs, C.I.s, and the p-values for all the assumed genetic modes of inheritance can be found in Table 3. 

## 4. Discussion

In the current study, we hypothesized that CD33 rs3865444 may have an impact on the cardinal functions of microglia and macrophages in MS pathophysiology and thus possibly lead to MS. To test this hypothesis, we genotyped a relatively large cohort of patients with MS for the CD33 rs3865444 variant. Our results showed that CD33 rs3865444 significantly influenced the risk of MS, as we observed a statistically significant association the variant and MS. More precisely, we presume that this difference in MS risk stems from either the GG (as a risk factor) or the GT (as a protective factor) genotype of CD33 rs3865444, as the GG one was more frequent, and the GT one was less frequent in MS patients compared to controls, while the observed frequency of the TT genotype did not differ between the two groups. As such, in this study, we provide preliminary indication for an influence of CD33 rs3865444 on MS.

One cannot argue against the contribution of genetic factors to MS susceptibility, as studies regarding the genetics of MS have come a long way in the last few years, and quite a few loci have been unequivocally identified [2,28,29,30,31]. Results from candidate-gene association studies and genome-wide association studies (GWAS) suggest that both common, low-frequency, and rare variants contribute to MS susceptibility [4,8]. Moreover, the variants that appear to have the largest effect on MS are the HLA DRB1*15:01 (as a risk factor) and the HLA A*:02 (as a protective one) haplotypes [4,28]. Besides them, over 200 non-HLA genetic variants polymorphisms have been reported to have some effect on MS, as well [32,33]. Additionally, some of the identified variants appear to not only alter the MS risk but also be associated with several MS endophenotypes [4,34]. Finally, several MS-associated variants are found in expression quantitative trait loci (eQTL) regions (in other words, genome regions with DNA sequence variants that influence the expression level of one or more genes) in different immune cells [2,35]. These data reflect the complexity of MS genetics and highlight the need for more research on this aspect of MS.

Besides genetics, a broad range of environmental factors further modify MS risk, including smoking, low vitamin D levels, Epstein–Barr virus (EBV) infection, adolescent obesity, oral tobacco use, alcohol consumption, cytomegalovirus (CMV) infection, coffee intake, night-shift work, and exposure to organic solvents [1]. As such, interactions between genetic, epigenetic [36,37] and environmental risk factors may predispose an individual towards developing MS [38].

The CD33 gene encodes the CD33 immune cell surface antigen. CD33 is exclusively expressed by microglia and macrophages (which are myeloid cells) in the brain [14,39]. While CD33 is mainly expressed on the myeloid lineage, it can also be found in some lymphoid lineage cells [13]. As such, CD33 is thought to be implicated in many pivotal processes of inflammation and immunity, affecting both myeloid and lymphoid lineages, and therefore alterations of CD33 may predispose to MS. Disease-modifying therapies for MS largely target the adaptive immune system, but these agents appear to have some important effects on myeloid cells as well [40]. Consequently, CD33 could be an attractive target mainly for myeloid but also for lymphoid cells. This is of particular interest as CD33 has been considered a therapeutic target for myeloid malignancies [41], using either anti-CD33 monoclonal antibodies (gemtuzumab ozogamicin, lintuzumab, and vadastuximab talirine) or gene-edited stem cells [39,41,42,43,44].

Recently, a frameshift variant (a 19-base pair deletion) in the CD33 gene has emerged as a potential risk locus for NMOSD [18]. More precisely, this 19-base pair deletion variant (chr19:51729579e51729597delCCAACAACTGGTATCTTTC), located exon 4 of the CD33 gene, results in a frameshift mutation with premature truncation (c.712-730delCCAACAACTGGTATCTTTC, p.Pro238Glnfs*38), leading to nonsense-mediated decay or to a shorter protein lacking 30 C-terminal amino acids [18]. This is intriguing, given that NMOSD and MS share some similarities despite the fact that they are two distinct diseases [19]. More precisely, microglial infiltration and lipid-laden macrophages, despite not being a landmark of the disease, have been reported in NMOSD [45]. Another similarity between NMOSD and MS is the cytokine profile, as recent studies suggest that the pathophysiology of both diseases includes Th17-mediated pathways, mainly responsible for demyelination [19,46]. However, how and to what extent CD33 affects the immune system and possibly contributes to these diseases can only be speculated at the moment.

From a genetic point of view, the common promoter variant CD33 rs3865444, the subject of this study, appears to modify, along with other genetic variants, the function of CD33 and has also been previously associated with AD. More precisely, the CD33 rs3865444 G risk allele for AD has been associated with higher CD33 cell surface expression, while the minor allele (found to be protective against AD) probably leads to an alternatively spliced CD33m isoform, lacking the Ig V-set domain encoded by exon 2 [39,47,48]. Therefore, we could assume that the CD33 rs3865444 GG genotype predisposes towards MS by increasing CD33 cell surface expression and decreasing the alternatively spliced CD33^m^ variant, thus altering myeloid cells function. 

The role of the CD33 gene in MS has not been fully elucidated yet. In order to validate our results, we searched for data from GWAS in MS, with complete summary statistics available in the GWAS catalog (https://www.ebi.ac.uk/gwas/efotraits/EFO_0003885) [49,50]. More precisely, we retrieved results for CD33 variants genotyped IN previous GWAS. We searched for CD33 variants in high linkage disequilibrium (LD) (r^2^ > 0.8) with the one genotyped in our study (CD33 rs3865444), namely, rs34813869, rs7245846, rs35408798, rs34912427, rs33978622, rs1354106, rs12459419. Among them, an LD proxy to CD33 rs3865444, i.e., rs1354106, has been identified, without it being associated with MS (OR = 0.9822, *p* = 0.3435). Based on this, the matter of whether CD33 rs3865444 is a common variant that has a small effect on MS genetic susceptibility is far from concluded.

Finally, the limits of our study must be mentioned. Firstly, our results should be overall interpreted with caution, given their modest ORs (≈ 0.8) compared with those from previous candidate gene association studies [33] or with those from conventional hypothesis-free GWAS [51], applying strict significance thresholds [52]. The deviation from HWE in MS cases (but not in controls) may be explained by the possible genetic association between the CD33 rs3865444 and MS [53]. Moving on, additional analysis for a possible association between CD33 rs3865444 and other outcomes (e.g., age at MS onset, response to treatments, multiple sclerosis severity score (MSSS) and expanded disability status scale (EDSS), annualized relapse rate (ARR), smoking behavior, etc.) could have given more robustness to our results. Moreover, the adjustment for other potential genetic risk factors for MS (especially, the HLA DRB1*15:01 and HLA A*:02), as well as the selection of genotyped variants based on the tagging SNP method, could have provided a comprehensive picture of the CD33 gene and its role in MS. Additionally, the possibility of loss of efficiency [54] (as the MS cases were almost triple the healthy control cases) cannot be completely overruled. Finally, replication in a second independent sample would have strengthened the validity of our findings.

## 5. Conclusions

In conclusion, we provide a preliminary indication for a possible effect of CD33 rs3865444 on the risk of MS. Whether CD33 rs3865444 can be considered a genetic risk factor for MS is far from concluded. Therefore, larger multiethnic studies should be conducted, investigating the role of CD33 rs3865444 in MS. Furthermore, analysis of other CD33 variants should be performed, ideally with the tagging SNP approach covering the entire variability of the CD33 gene. For future studies, focus should be placed on CD33 expression in the CNS of MS patients, along with microglia activation and overall CD33 gene variability.

## Figures and Tables

**Table 1:** Demographic and clinical characteristics of the study participants.

	Healthy Controls	MS Patients
*n*	400	1396
Female, *n* (%)	257 (64.25)	868 (62.18)
Male, *n* (%)	143 (35.75)	528 (37.82)
Female/Male ratio	1.79	1.64
Age at time of analysis, mean (years)	40.12	39.17

MS, multiple sclerosis.

**Table 2:** Allelic and genotype frequencies of CD33 rs3865444 in healthy controls, in MS cases, and the whole sample.

SNP	Genotypes/Alleles	Healthy Controls (*n* = 400)	MS (*n* = 1396)	Whole Sample (*n* = 1796)
rs3865444		*n* (%)	*n* (%)	*n* (%)
Genotype	G/G	215 (0.54)	807 (0.60)	1022 (0.58)
	G/T	156 (0.39)	449 (0.33)	605 (0.35)
	T/T	27 (0.07)	94 (0.07)	121 (0.07)
Allele	G	586 (0.74)	2063 (0.76)	2649 (0.76)
	T	210 (0.26)	637 (0.24)	847 (0.24)

SNP, single-nucleotide polymorphism.

**Table 3:** Single-locus analysis for the association between CD33 rs3865444 and MS, in co-dominant, dominant, recessive, over-dominant, and log-additive modes.

Mode	Genotype	OR (95% CI)	*p*-Value
Co-dominant	G/G	1.00	0.089
	T/G	0.77 (0.61–0.97)	
	T/T	0.93 (0.59–1.46)	
Dominant	G/G	1.00	0.041
	T/G-T/T	0.79 (0.63–0.99)	
Recessive	G/G-T/G	1.00	0.9
	T/T	1.03 (0.66–1.60)	
Over-dominant	G/G-T/T	1.00	0.03
	T/G	0.77 (0.61–0.97)	
Log-additive	---	0.87 (0.73–1.04)	0.12

CI: confidence interval; OR: odds ratio. Statistically significant values are presented in bold font.
